# Dynamic Ferromagnetic Hysteresis Modelling Using a Preisach-Recurrent Neural Network Model

**DOI:** 10.3390/ma13112561

**Published:** 2020-06-04

**Authors:** Christian Grech, Marco Buzio, Mariano Pentella, Nicholas Sammut

**Affiliations:** 1 Faculty of Information and Communications Technology, University of Malta, MSD2080 Msida, Malta; nicholas.sammut@um.edu.mt; 2 CERN, European Organization for Nuclear Research, 1211 Geneva, Switzerland; Marco.Buzio@cern.ch (M.B.); mariano.pentella@cern.ch (M.P.); 3 Department of Applied Science and Technology, Politecnico di Torino, 10129 Turin, Italy

**Keywords:** ARMCO pure iron, dynamic hysteresis loop, machine learning, magnetic properties, particle accelerators, Preisach, recurrent neural networks

## Abstract

In this work, a Preisach-recurrent neural network model is proposed to predict the dynamic hysteresis in ARMCO pure iron, an important soft magnetic material in particle accelerator magnets. A recurrent neural network coupled with Preisach play operators is proposed, along with a novel validation method for the identification of the model’s parameters. The proposed model is found to predict the magnetic flux density of ARMCO pure iron with a Normalised Root Mean Square Error (NRMSE) better than 0.7%, when trained with just six different hysteresis loops. The model is evaluated using ramp-rates not used in the training procedure, which shows the ability of the model to predict data which has not been measured. The results demonstrate that the Preisach model based on a recurrent neural network can accurately describe ferromagnetic dynamic hysteresis when trained with a limited amount of data, showing the model’s potential in the field of materials science.

## 1. Introduction

The phenomenon of hysteresis occurs when a system is not only dependent on present input values, but also on past input values. The resulting hysteresis loops make modelling a challenge due to the non-linearity exhibited. In phenomena such as ferromagnetism, dynamic effects add a further aspect of complexity. The interaction between electric and magnetic fields results in induced eddy currents, which modify the magnetic hysteresis characteristics. Proposed models to predict hysteresis can be classified into two main categories: physical models and phenomenological models. The physical models are built on the description of the object being modelled using physics laws. Unfortunately, in most cases such models are mathematically complex and require detailed knowledge of the physical properties of the material being modelled. Phenomenological models make use of conventional identification methods, which typically have no physical meaning. Such models are mostly empirical, based on previously acquired experimental data. The most popular phenomenological hysteresis model is the Preisach model [1], used in a vast range of applications. Among the different hysteresis models available in literature, the Preisach-type models prove to have a great potential to explain various magnetization processes, and is one of the most popular models to capture the hysteresis behaviour in non-linear systems.

The biggest challenge in implementing a Preisach model is the identification of the model parameters, because the use of empirical methods makes the procedure problem-dependent [2]. There are numerous identification methods used in literature [3,4,5]. In most cases, such a function can be identified using measured data and applying a numerical approach, which can be a specific formulation [6,7] or using conventional methods such as an Everett integral [8] or Gaussian/Lorentzian functions [9]. The function can then be applied in software using a look-up table of values. Other ‘black-box’ identification techniques used alongside the Preisach model include genetic algorithms [10], fuzzy models [11] and artificial neural networks (ANN) [12,13,14]. In [15], Saliah et al. showed that using an ANN can match results produced by the conventional methods, considerably reducing the time overhead. In [16], Serpico and Visone build an ANN hysteresis model, which is able to model rate-independent hysteresis when combined with Preisach operators as inputs. Similarly, different neural network configurations have been used for modelling the rate-independent hysteresis of magnetic shape memory alloys [17,18].

The Preisach model implementation described above is rate-independent, meaning that the hysteresis output is determined solely by the input’s extreme values, and the input ramp rates do not impact the hysteresis loop. Mayergoyz [19] introduced the dependence of the weight function on the speed of output variations; similarly, Mrad and Hu [20] and Song and Li [21] proposed an input-rate dependence of the weight function. Both methods suppose the weight function is the right place to add in dynamic behaviors. On the other hand, in [22] a linear dynamic model is added before the classical Preisach operator and the dynamics are assumed to only happen inside the linear dynamic part. The latter cascade structure can be referred to as an ‘external dynamic hysteresis model’ and is found in several works [23,24,25]. In this work, we will consider the rate-dependency without including an additional element. This can be accounted for by implementing the Preisach model’s weighting function using dynamic neural networks such as a recurrent neural network (RNN), where each layer has a recurrent connection, allowing the network to have an infinite dynamic response to time series input data. An example of the implementation of such model configurations for hysteretic data can be found in [26], where an Elman RNN was successful in predicting the major loop at several frequencies. In [27], with the addition of Preisach operators at the input stage of an internal time-delay neural network, the major and minor loop hysteresis of a Giant Magnetostrictive Actuator was modelled successfully, however this work did not demonstrate functionality in the saturation regions.

In this paper, a Preisach model is implemented using a single RNN, which is able to predict the different dynamic hysteresis loops of ferromagnetic materials, when a limited amount of measurement data is available. In particular, the model is trained using three particular frequencies and tested on a data set which consists of a different frequency. As a novel contribution to the current research state, both major and minor loop hysteresis are investigated, including saturation regions. The motivation behind this work lies in the prediction of the dynamic behaviour of materials used in the manufacturing of magnets for particle accelerators. As an example, ARMCO is used as a test material being used as part of the superconducting magnets for the High Luminosity upgrade of the Large Hadron Collider at the European Nuclear Research Centre (CERN) [28,29], where pulsed fields are employed. Hence, the knowledge of the material’s dynamic behaviour, which is not easily modelled, is required and this work attempts to propose an accurate model, trained with a limited amount of measurement data, which can also be used at a moderate computational cost. Whilst a vector hysteresis model is required for a 3D model of an accelerator magnet, the model can be used to describe the vertical field used for the RF control of synchrotrons. The description of the eddy-currents transients when ramping up the material during a test is complicated due to its hysteretic behaviour and the geometry, generally toroidal, which complicates the formulation [30]. This combined with the intrinsic nature of the flux-metric method adopted for the material measurement, introduces an uncertainty component on the results, especially on the coercive field determination [31,32]. The knowledge of the dynamic behaviour of the material can be potentially used to extrapolate the data in DC, allowing the separation of the rate-independent hysteresis from the rate-dependent part, and reducing the overall uncertainty.

In Section 2, the experimental details behind the measurements, the theory behind the model and the validation technique are described. Section 3 describes the results obtained, including the results of a univariate sensitivity analysis. As test material, ARMCO was considered, being an important yoke material in particle accelerators [33]. Moreover, having an electrical conductivitiy in the order of 10${}^{8}$ S/m and a relative permeability in ther order of 3000, the effect of the eddy currents on the hysteresis loop can be measured with a good signal-to-noise ratio.

## 2. Materials and Methods

### 2.1. Experimental Details

**Figure 1:**
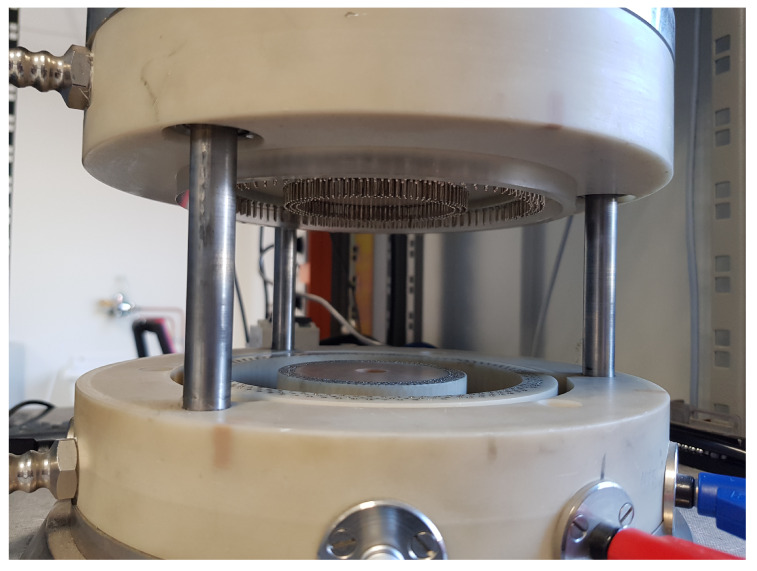
The split-coil permeameter.

The measurements in this work are performed using a split-coil permeameter [28], shown in Figure 1, and performed on toroidal test specimens. The equipment consists of three 90-turn coils, separable by means of an opening mechanism. A slot allows the insertion of the sample to be tested in the permeameter, therefore avoiding the time-demanding operation of a custom coil winding onto the sample [34]. The two outermost coils are used as excitation coils and powered in series up to a maximum current of 40 A, corresponding to a maximum magnetic field of 24 kAm${}^{-1}$. The innermost coil is used to detect the induced voltage. The entire system is cooled by compressed air.

**Figure 2:**
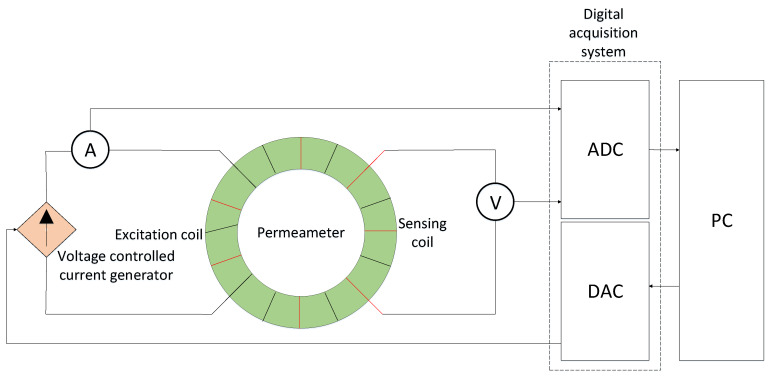
Measurement system layout.

The measurements are performed by means of the fluxmetric method, as shown in Figure 2. The test specimen is magnetized by the two excitation coils, having in total $N_{e}$ = 180 turns and powered in series by a voltage-controlled current generator. Given $r_{1}$ and $r_{2}$ respectively the inner and the outer radius of the test specimen, the magnetic field is equal to:

(1) $$H\left(t\right)=\frac{N_{e}i\left(t\right)}{2\pi r_{0}}$$

where:

(2) $$r_{0}=\frac{r_{2}-r_{1}}{ln\frac{r_{2}}{r_{1}}}$$

The magnetic flux density is evaluated by:

(3) $$B\left(t\right)=\frac{1}{A_{s}}\left(\frac{\Phi (t)}{N_{s}}-\mu_{0}H\left(A_{t}-A_{s}\right)\right)$$

where $A_{s}$ is the cross-sectional area of the sample, $A_{t}$ the cross-sectional area of the sensing coil, $N_{s}$ is the number of turns of the sensing coil, $\mu_{0}$ the permeability of the free space and Φ(t) the magnetic flux, evaluated by integrating the induced voltage.

Both the current and the voltage are acquired by a digital acquisition system (DAQ), a NI 4461 [35] by National Instruments at frequency of 20 kHz. In particular, the value of the current is acquired using a MACC${}^{2}$ PLUS direct current transducer [36]. The value of the magnetic flux density is acquired with an uncertainty of 0.5 mT whereas the magnetic field is known with an accuracy of 0.1 Am${}^{-1}$. The current is ramped back and forth, between positive and negative symmetric values, and at the end of each ramp the current is kept constant for 0.5 s.

For the major hysteresis loops, the plateau amplitudes are chosen in such a way that the material is brought in saturation state. In order to acquire only the hysteresis loop without including the initial magnetization branch, a pre-cycle is applied before the specific cycle. In the case of the minor loops, the same procedure is carried out, with the difference that the plateau amplitudes at each cycle are increased. Between each acquisition and the following one, the sample is demagnetized. The magnetic flux density, B(t) in different dynamic hysteretic conditions obtained using the magnetic field, H(t) are the source of information for this work. In the case of minor loops, this work is limited to symmetrical, first-order curves.

### 2.2. Hysteresis Modelling Based on Preisach Memory

**Figure 3:**
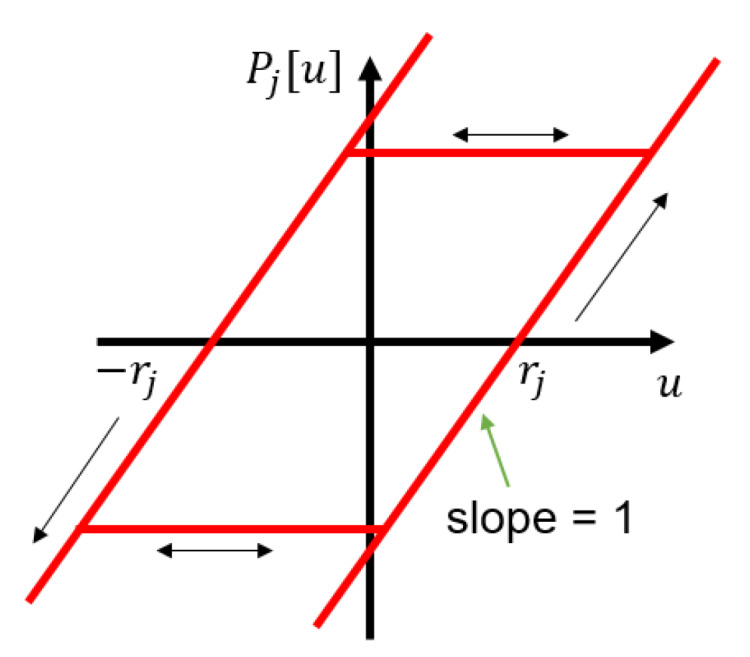
Play operator.

In general, the Preisach model is expressed using a double integrator in continuous form as [1]:

(4) $$\hat{y}\left(t\right)=\underset{\alpha \geq \beta }{\int \int }\mu \left(\alpha ,\beta \right)\gamma_{\alpha \beta }u\left(t\right)d\alpha d\beta$$

where $\hat{y}\left(t\right)$ is the model output at time *t*, u(t) is the model input at time *t*, while $\gamma_{\alpha \beta }$ are elementary rectangular hysteresis operators with α and β being the up and down switching values, respectively. These operators can only assume a value of +1 or −1. The density function μ(α,β) is a weighting function, which represents the only model unknown which has to be determined from experimental data. In [37,38], following a change in co-ordinates r=(α−β)/2, v=(α+β), $\hat{\mu }=\mu \left(v+r,v-r\right)$, it is shown that the boundary between the +1 and −1 regions in the Preisach half-plane with coordinates r>0,v∈ℝ, is described by the function v=P[u](t), known as the play operator. This makes it possible to rearrange Equation (4) as:

(5) $$\hat{y}\left(t\right)=\int_{0}^{+\infty }g\left(r,P\left[u\left(t\right)\right]\right)\;dr$$

which can be discretized to *n* play operators as follows:

(6) $$\hat{y}\left(t\right)=\sum_{j=1}^{n}\phi_{j}P_{j}\left[u\right]\left(t\right)$$

where $\phi_{j}$ represents the density function of the *j*th play operator, which has to be identified. The play operator is shown in Figure 3, and defined in Equation (7):

(7) $$\begin{array}{cc} P_{j}\left[u\right]\left(t\right) & =\max(u\left(t\right)-r_{j},\min\left(u\left(t\right)+r_{j},P_{j}\left[u\right]\left(t-1\right)\right)) \end{array}$$

(8) $$\begin{array}{cc} P_{j}\left[0\right] & =\max(u\left(0\right)-r_{j},\min\left(u\left(0\right)+r_{j},k_{0}\right)) \end{array}$$

where $k_{0}$ is the initial condition of the operator and $r_{j}$ represents the memory depth as follows:

(9) $$r_{j}=\frac{j-1}{n}\left[\max\left(u\left(t\right)\right)-\min\left(u\left(t\right)\right)\right]$$

where j=1,2,3,⋯n.

### 2.3. Identifying ϕ Using Recurrent Neural Networks

Artificial neural networks are able to map non-linear data in various applications. In this case, a recurrent neural network (RNN) will be used to replace the density function $\phi_{j}$ of the discrete Preisach model (Equation (6)), as these structures are recognized for their ability to model any non-linear dynamic system, up to a given degree of accuracy [39]. RNNs are distinguished from feed-forward networks by the feedback loop connected to their past decisions, ingesting their own outputs moment after moment as input. This means that such networks can be used to model dynamic characteristics. Sequential information is preserved in the recurrent network’s context layer, which manages to span many time steps as it cascades forward to affect the processing of each new example.

**Figure 4:**
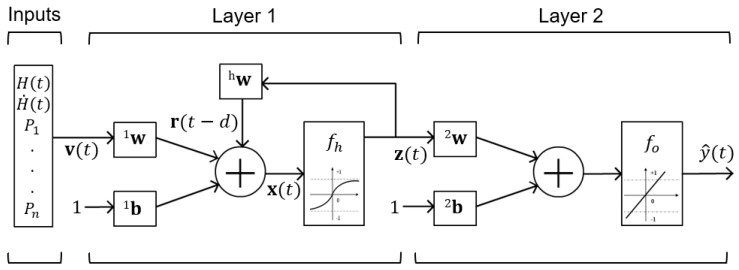
Schematic diagram of the Preisach-RNN model.

In general, a RNN can be seen as a group of nodes, consisting of three different kinds of nodes, namely the input, hidden and output nodes, organized in separate layers, as shown in Figure 4. While the input and output layers consist of feed-forward connections, the hidden layer has recurrent ones. At each time step, *t*, the input vector v(t) is processed at the input layer. Each instant v(t) is summed with the bias vector ${}^{1}b$ and multiplied by the input weight matrix ${}^{1}w$. Analogously, the internal state z(t), delayed by a number of time instants *d*, is multiplied by the gain factor ${}^{h}w$ and added to the input state as follows:

(10) $$z\left(t\right)=f_{h}\left[{}^{1}w\left(v\left(t\right)+{}^{1}b\right)+{}^{h}w\left(z\left(t-d\right)\right)\right]$$

where $f_{h}\left(x\right)$ is the activation function, in this case a hyperbolic tangent function, which is given as:

(11) $$f_{h}\left(x\right)=\frac{2}{1+e^{-2x}}-1$$

The internal state z(t) is then added with bias ${}^{2}b$, multiplied by the weight ${}^{2}w$, and the result is passed through a linear activation function $f_{o}\left(x\right)$ as follows:

(12) $$\hat{y}\left(t\right)=f_{o}\left[{}^{2}w\left(z\left(t\right)+{}^{2}b\right)\right]$$

where $\hat{y}\left(t\right)$ is the predicted output at time *t*.

The Deep Learning Toolbox [40] by MATLAB is used to determine the weights of the network using the *layrecnet* command. The training algorithm is the Levenberg-Marquadt algorithm [41], which is a non-linear least squares optimization algorithm incorporated into the backpropagation algorithm for training neural networks as demonstrated in detail in [42]. The algorithm aims to optimize the weights according to the following objective function:

(13) $$V\left(t\right)=\frac{1}{2}{\left(y\left(t\right)-\hat{y}\left(t\right)\right)}^{T}\left(y\left(t\right)-\hat{y}\left(t\right)\right)$$

which leads to the update of the weights by the following formula:

(14) $${}^{i}w\left(t+1\right)={}^{i}w\left(t\right)+\eta \left(-\frac{\partial V}{\partial t}\right)$$

where η is a positive number representing the learning rate of the weights.

### 2.4. Data Analysis Approach

The input data to the model include the magnetic field H(t), its derivative $\dot{H}\left(t\right)$ and a number of play operators $P_{j}\left[H\right]\left(t\right)$ (chosen according to the complexity of the data). All data is normalised to the range of the minor loop data, such that data is approximately in the range [−1,1]. The play operator outputs, $P_{j}$, are subsequently calculated based on the normalised magnetic field signal. In this work, the predicted variable is the magnetic flux density measured in Section 2.1, and all measurements are downsampled to 200 Hz.

**Figure 5:**
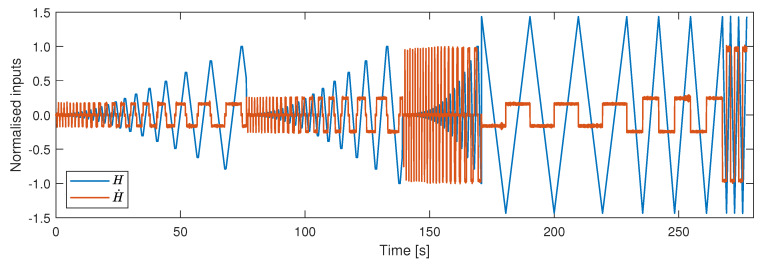
Magnetic field data and its derivative used for training, validation and testing the model.

**Figure 6:**
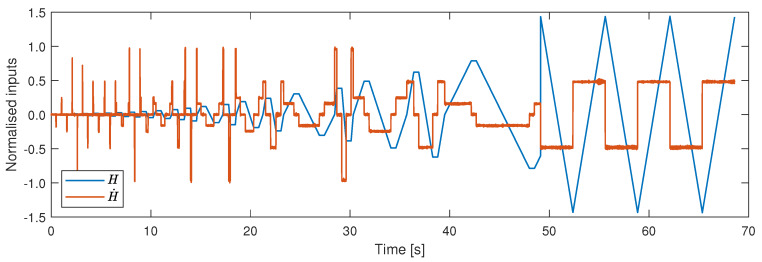
Magnetic field data and its derivative used for evaluating the model.

The data set used for training, validation and testing comprises of three minor loops and three major loops ramping at 1025, 1554 and 6135 Am${}^{-1}$s${}^{-1}$, as shown in Figure 5. This data is divided into three subsets in an interleaved manner. The first subset is the training set (70% of the data), which is used for updating the network weights and biases. The second subset is the validation set (15% of the data), used to decide when to stop training the model, whilst the final set is the testing set (15% of the data), which is used to select the best model structure. Once the network is trained, and the best model structure is chosen, an evaluation signal is used to demonstrate the performance of the model. The evaluation data set consists of three major loops ramping at 3067 Am${}^{-1}$s${}^{-1}$ and one minor loop ramping at various random ramp-rates between 1000 and 6200 Am${}^{-1}$s${}^{-1}$, as shown in Figure 6.

### 2.5. Model Validation

**Figure 7:**
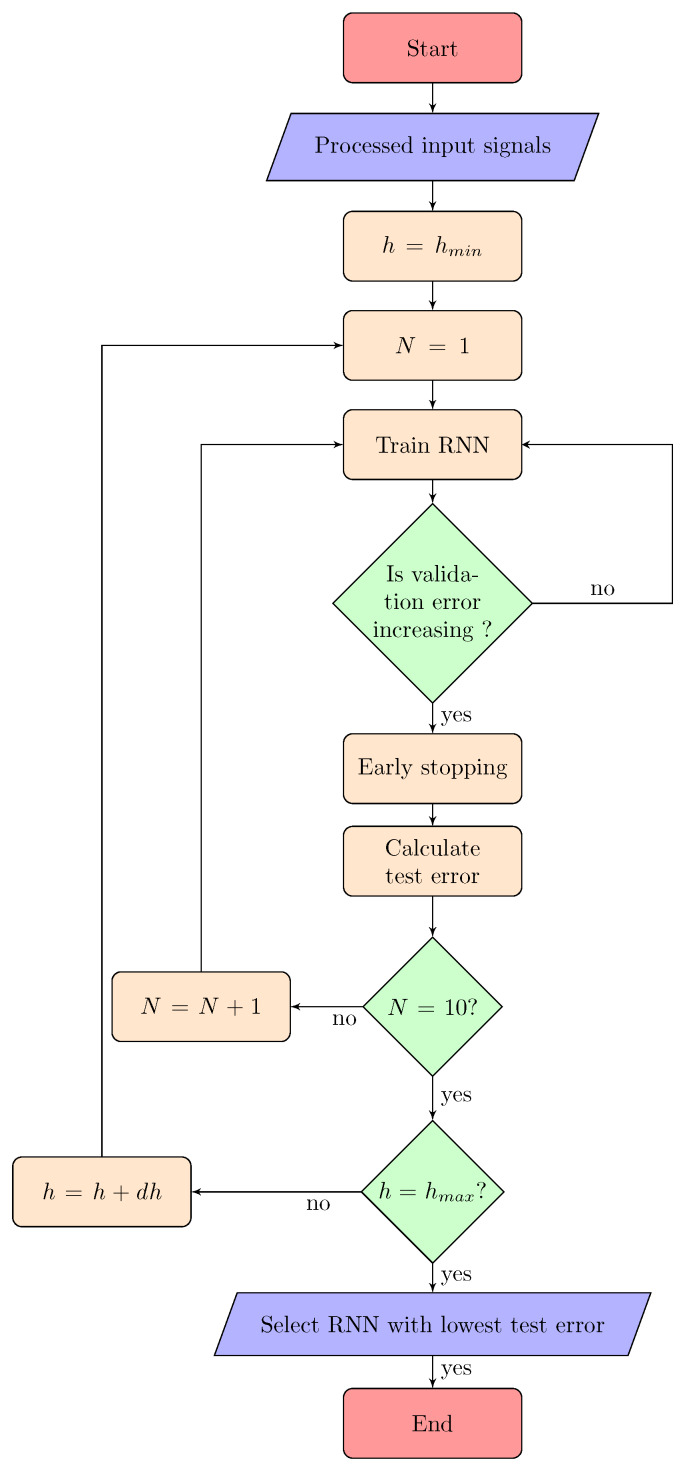
Flowchart showing the model training and validation procedure.

Model validation is the process of choosing the best model parameters and making sure that the model is robust to new data. The optimal number of nodes in the hidden layer, *h* is chosen by doing a search over a range of values. The complete validation process explained below consists of three loops, and is represented by a flowchart in Figure 7.

In the innermost loop, each neural network is trained using the training set as described in Section 2.3 over a certain number of repetitions called epochs. The number of epochs is determined using a method called early stopping [43,44]. In this technique, the error on the validation set is monitored during the training process. The validation error normally decreases during the initial phase of training, as does the training set error. However, when the network begins to overfit the data, the error on the validation set typically begins to rise. When the validation error increases for a specified number of iterations, the training is stopped, and the weights and biases at the minimum of the validation error are returned. Finally, the testing set is used to obtain the performance of the particular network.

Each time a neural network is trained, a different solution is obtained due to different and random initial weight and bias values. As a result, different neural networks trained on the same problem can give different outputs for the same input. To ensure that a neural network of good accuracy has been found, each network is retrained for a number of times, *N*, which in this work is 10.

In the outermost loop, the number of hidden nodes is varied over the range starting from $h_{min}$ to $h_{max}$ with increments of dh. The network with the best test performance having a number of hidden nodes *h*, is hence chosen and used with the evaluation data set. The complete list of parameters used in this work is given in Table 1.

### 2.6. Performance Indicator

The performance indicator used in this work to represent the error between the model and experimental measurements is the normalised root mean square error (NRMSE):

(15) $$\mathrm{NRMSE}\left(y,\hat{y}\right)=\frac{1}{\max(y)-\min(y)}\sqrt{\frac{1}{N}\sum_{i=1}^{N}{\left(y_{i}-\hat{y}\right)}_{i}^{2}}$$

where *y* is the actual value, $\hat{y}$ is the modelled quantity and *N* is the number of samples considered. By normalising the root mean square error, the errors can be scaled relatively to the range of the measurement, thus allowing an appropriate comparison across different conditions.

## 3. Results

Results from the model training, testing and sensitivity analysis are presented and discussed in this section.

### 3.1. Minor and Major Loop Model Prediction

**Figure 8:**
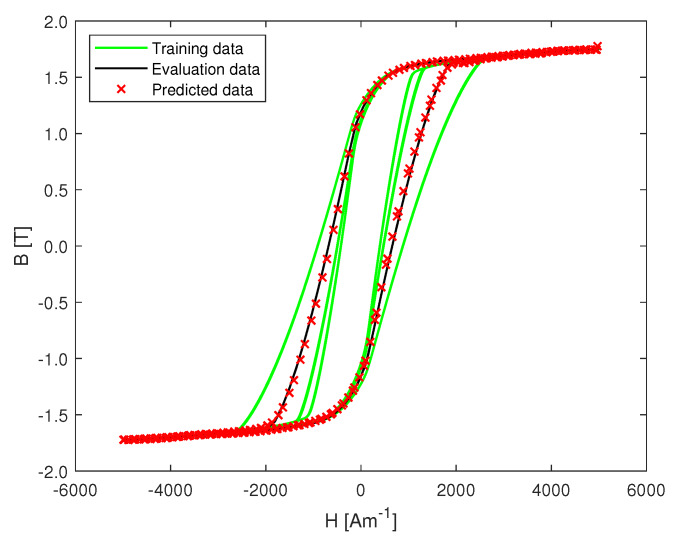
Predicted major loop and experimental data used in training the model.

**Figure 9:**
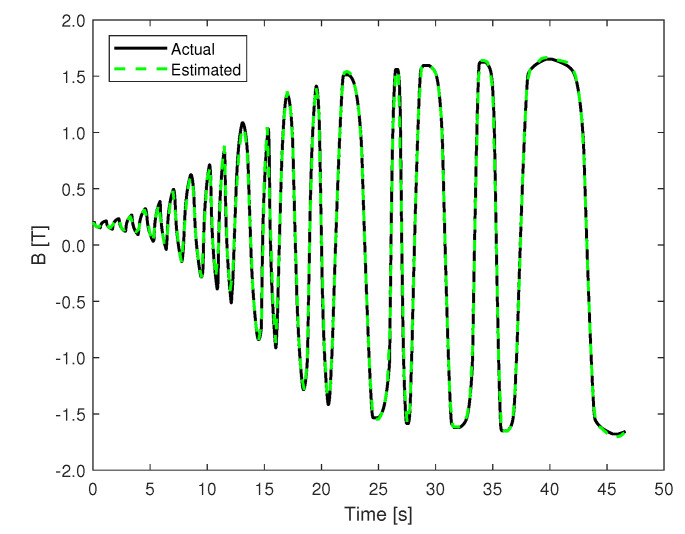
Predicted minor loop with random ramp-rates.

Following the model validation and training procedure explained in the previous section, the best performing model is used for predicting the data in the evaluation set shown in Figure 6. In order to get rid of the initial transient phase of the model, the first few predicted samples in the set are discarded. In the case of the major loop, a NRMSE of 0.58% is noted in predicting a hysteresis loop with a new ramp-rate not used for training. Figure 8 shows the major loop data used for training and evaluating the model, as well as the predicted data. For the minor loop data, a NRMSE of 0.66% is noted with different random ramp-rates, including ramp-rates not used for training the model. The magnetic flux density as a function of time is shown in Figure 9.

### 3.2. Effect of Preisach Operators on Performance

In order to quantify the impact of Preisach operators as part of the model, the model’s training and validation procedure is repeated without the $P_{j}$ inputs. Comparing the performance of the two models, the inclusion of the Preisach operators is noted to improve performance by 19% in the case of major loop hysteresis, and 44% in the case of minor loop hysteresis. It has to be noted however, that a model without Preisach operators is computationally less expensive, as the optimal structure contains 241 weights versus 409 weights in the original model. Hence, in the end, a compromise must be made between accuracy and speed according to the model’s application.

### 3.3. Univariate Sensitivity Analysis

A univariate sensitivity analysis provides information on how robust a model is when the input values are varied over a specific range [45]. This analysis is performed to understand which model input variables impact the prediction most significantly, especially since the magnetic field derivative signal, $\dot{H}\left(t\right)$ is noisy. A neural network which picks up minor noise during training can overfit the noise as if it is the signal, leading to poor accuracy during validation [46]. One way to confirm this is by performing a sensitivity analysis, where one input parameter is fed a changing signal whilst keeping the other inputs constant, and checking the deviation in the output signal. This is repeated for all the model inputs.

**Figure 10:**
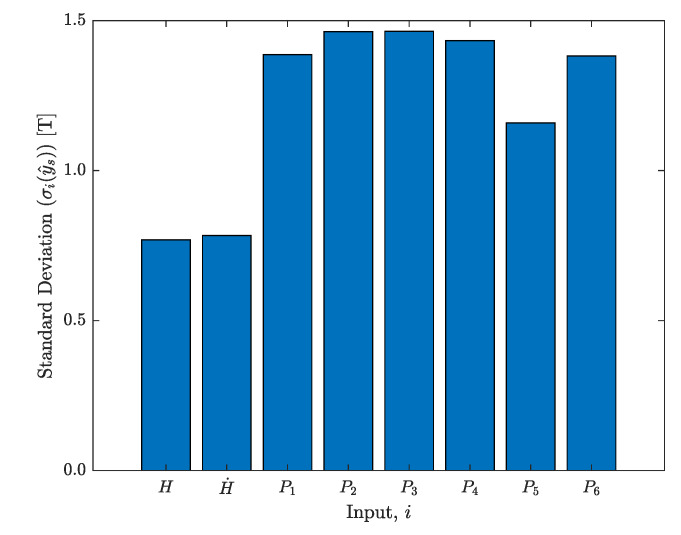
Sensitivity analysis results for each individual input.

In this exercise, once the model is trained, an input vector is defined and set as 0. Then, for one of the eight variables, a value is assigned between the [−1,1] range. This is repeated for 10,000 samples for each input variable. The predicted output, ${\hat{y}}_{s}$ is saved and the standard deviation $\sigma_{i}\left({\hat{y}}_{s}\right)$ for each variable *i* is calculated. The bar chart in Figure 10 illustrates these results. The analysis results show a higher variation in the predicted output for play operator input parameters, thus the model is more sensitive to changes in these particular variables. These results also confirm the fact that even though the model is trained with a noisy $\dot{H}$ signal, the predicted output is not particularly sensitive to perturbations from this parameter.

## 4. Conclusions

A Preisach-RNN model is proposed to predict the dynamic characteristics of ferromagnetic materials that does not require *a priori* knowledge of the material and its microstructural behaviour. The model is based on the Preisach memory block where the density function is represented by a recurrent neural network. A thorough training and validation procedure is proposed for the neural network, in order to optimize the weight parameters. We have demonstrated, using ARMCO pure iron measurements, that such a model can predict both major and minor loop dynamic ferromagnetic hysteresis behaviour allowing researchers to estimate the dynamic effects of the material knowing only six different examples at three frequencies. Comparing the model’s predictions to experimental data, the model’s NRMSE is noted to be better than 0.7%. Moreover, these results prove that the model generalises well to new data, and can potentially be used at different frequencies than the frequencies used in this work. In validating the performance of the model, the positive impact of Preisach operators is noted, even though this comes at a computational cost, which has to be evaluated for each specific industry. Results from a univariate sensitivity analysis also demonstrate that the play operator inputs impact the predicted magnetic flux output most significantly. We believe that this model, predicting major and minor loop hysteresis under different dynamic conditions can be applied to other dynamic hysteresis prediction problems in the realm of materials science.

## Figures and Tables

**Table 1:** RNN parameters list.

	Parameter	Value
Model properties	Activation function (hidden layer)	sigmoid
	Activation function (output layer)	linear
	delay, *d*	2
	hidden nodes search range, $h_{min}:dh:h_{max}$	4:1:13
	number of hidden nodes, *h*	12
	training repetitions, *N*	10
Input	No. of play operators	6
Training set	data	70% of data set
	epochs	≤1000
	algorithm	Levenberg-Marquadt
Validation set	data	15% of data set
Testing set	data	15% of data set
Evaluation set	data	unseen data
	metric	NRMSE

## Abbreviations

The following abbreviations are used in this manuscript:

ANN	Artificial Neural Network
CERN	European Organization for Nuclear Research
NRMSE	Normalised Root Mean Square Error
RNN	Recurrent Neural Network
